# Novel mutations in human and mouse *SCN4A* implicate AMPK in myotonia and periodic paralysis

**DOI:** 10.1093/brain/awu292

**Published:** 2014-10-20

**Authors:** Silvia Corrochano, Roope Männikkö, Peter I. Joyce, Philip McGoldrick, Jessica Wettstein, Glenda Lassi, Dipa L. Raja Rayan, Gonzalo Blanco, Colin Quinn, Andrianos Liavas, Arimantas Lionikas, Neta Amior, James Dick, Estelle G. Healy, Michelle Stewart, Sarah Carter, Marie Hutchinson, Liz Bentley, Pietro Fratta, Andrea Cortese, Roger Cox, Steve D. M. Brown, Valter Tucci, Henning Wackerhage, Anthony A. Amato, Linda Greensmith, Martin Koltzenburg, Michael G. Hanna, Abraham Acevedo-Arozena

**Affiliations:** 1 MRC Mammalian Genetics Unit, Harwell, Oxfordshire, UK; 2 UCL Institute of Neurology, MRC Centre for Neuromuscular Diseases, London, UK; 3 University of Aberdeen, Institute of Medical Sciences, Scotland, UK; 4 Department of Neuroscience and Brain Technologies, Istituto Italiano di Tecnologia, Genova, Italy; 5 Biology Department, University of York, York, UK; 6 Brigham and Women's Hospital, Harvard Medical School, Boston, US; 7 Mondino National Institute of Neurology Foundation, IRCCS, Pavia, Italy

**Keywords:** SCN4A, mice, AMPK, periodic paralysis, myotonia

## Abstract

Mutations in the skeletal muscle channel (*SCN4A*), encoding the Na_v_1.4 voltage-gated sodium channel, are causative of a variety of muscle channelopathies, including non-dystrophic myotonias and periodic paralysis. The effects of many of these mutations on channel function have been characterized both *in vitro* and *in vivo*. However, little is known about the consequences of *SCN4A* mutations downstream from their impact on the electrophysiology of the Na_v_1.4 channel. Here we report the discovery of a novel *SCN4A* mutation (c.1762A>G; p.I588V) in a patient with myotonia and periodic paralysis, located within the S1 segment of the second domain of the Na_v_1.4 channel. Using *N*-ethyl-*N*-nitrosourea mutagenesis, we generated and characterized a mouse model (named draggen), carrying the equivalent point mutation (c.1744A>G; p.I582V) to that found in the patient with periodic paralysis and myotonia. Draggen mice have myotonia and suffer from intermittent hind-limb immobility attacks. In-depth characterization of draggen mice uncovered novel systemic metabolic abnormalities in *Scn4a* mouse models and provided novel insights into disease mechanisms. We discovered metabolic alterations leading to lean mice, as well as abnormal AMP-activated protein kinase activation, which were associated with the immobility attacks and may provide a novel potential therapeutic target.

## Abstract (precis)

Corrochano Sanchez *et al.* identify a novel mutation (I588V) in *SCN4A,* which encodes the Nav1.4 voltage-gated sodium channel, in a patient with myotonia and periodic paralysis. By generating and characterizing a mouse model (‘draggen’) carrying the equivalent point mutation (I582V), they uncover novel pathological and metabolic features of *SCN4A* channelopathies.

## Introduction

The voltage-dependent sodium skeletal muscle α-subunit Na_v_1.4 channel, encoded by the *SCN4A* gene, is responsible for the initiation and propagation of the action potential in the muscle fibres that results in muscle contraction. Other ion channels in muscle fibre membranes (potassium, calcium and chloride channels) control the resting membrane potential, the repolarization following an action potential, and the Ca^2+^ entry into the cytoplasm that govern the contraction and the relaxation of the muscles. There are many pathological mutations found in the *SCN4A* gene leading to changes in the electrophysiological properties of the Na_v_1.4 channel and in muscle fibre excitability (Cannon, 1996*a*, 2006; Webb and Cannon, 2008).

The Na_v_1.4 channel is a large protein composed of four homologous domains (I–IV), each with six transmembrane helices (S1–S6). The majority of causative *SCN4A* mutations are missense, gain of function mutations (Tsujino *et al.*, 2003; Jurkat-Rott *et al.*, 2010; Lossin *et al.*, 2012), and are usually located within the pore forming segments S5–S6, the voltage sensor segment S4 or the S4–S5 linker of the Na_v_1.4 channel. There are a number of clinically classified types of muscle channelopathies caused by mutations in the *SCN4A* gene (Vicart *et al.*, 2005), including: potassium-aggravated myotonia, paramyotonia congenita, hyperkalaemic periodic paralysis and hypokalaemic periodic paralysis. The defining features of *SCN4A* skeletal muscle channelopathies are myotonia and/or muscle weakness (Fontaine *et al.*, 1990; Cannon, 1996*b*). Periodic paralysis is characterized by reduced muscle membrane excitability, resulting in attacks of muscle weakness (Fontaine *et al.*, 1990; Rojas *et al.*, 1991; McClatchey *et al.*, 1992). The attacks are often associated with increased (hyperkalaemic periodic paralysis) or reduced (hypokalaemic periodic paralysis) serum potassium levels. Myotonia is in turn characterized by increased muscle membrane excitability, leading to muscle stiffness (Matthews *et al.*, 2010). There is some overlap in the clinical symptoms; for example patients diagnosed with hyperkalaemic periodic paralysis and paramyotonia congenita can present with attacks of both myotonia and paralysis. In a recent attempt to categorize patients with hyperkalaemic periodic paralysis, three clinical subgroups were proposed depending if patients presented without myotonia, with clinical or EMG myotonia or with paramyotonia congenita (Charles *et al.*, 2013). In addition, the same *SCN4A* mutation can be found in patients with different clinical diagnosis (Sasaki *et al.*, 1999; Okuda *et al.*, 2001; Jurkat-Rott and Lehmann-Horn, 2007). Overall, there is little known about the consequences of *SCN4A* mutations in the muscle downstream of their impact on the electrophysiology of the Na_v_1.4 channel.

In recent years two knock-in mouse models of periodic paralysis have been generated to study the effects of *Scn4a* mutations on skeletal muscle function. The first *Scn4a* knock-in model carries the mouse equivalent of the most common human hyperkalaemic periodic paralysis mutation (M1592V) (Hayward *et al.*, 2008). Heterozygous mice develop myotonia and progressive myopathy, together with *ex vivo* potassium-aggravated muscle weakness. The other knock-in strain models hypokalaemic periodic paralysis via another *Scn4a* mutation (R669H) (Wu *et al.*, 2011). More recently a *Cacna1s* knock-in model of periodic paralysis has been developed (Wu *et al.*, 2012).

Skeletal muscle is a major metabolic regulator where ATP homeostasis is critical for its function. Conversely, pathological alterations in skeletal muscle can lead to a disruption in the whole body metabolic balance (Pedersen and Febbraio, 2012). Muscles convert the chemical energy of carbohydrates and fats first into chemical energy (ATP) and then into mechanical work and heat. The use of energy in the form of ATP during muscle contraction leads to an increase in the concentrations of AMP and ADP that in turn activate AMP-activated protein kinase (AMPK) via phosphorylation at its Thr172 residue (Hardie and Sakamoto, 2006). As a consequence, active AMPK inhibits anabolic and activates catabolic pathways to increase the rate and capacity of ATP production (Winder and Hardie, 1996; O'Neill *et al.*, 2013). AMPK signalling is therefore regarded as a key regulator of energy status in muscle fibres. Ion channels control muscle contractile activity and thus can affect ATP turnover in muscle fibres. AMPK activation has been reported to regulate ion channel activity directly (Evans *et al.*, 2012) and indirectly (Light *et al.*, 2003), thus providing a link between cellular energy demands and ion channel activity (Ingwersen *et al.*, 2011; Andersen and Rasmussen, 2012).

Here we report the identification of a novel dominant *SCN4A* mutation identified in a patient presenting with myotonia and periodic paralysis. We also report the identification of the mouse equivalent of this mutation, which we named draggen, found in an *N*-ethyl-*N*-nitrosurea (ENU) mutagenesis screen (Nolan *et al.*, 2002; Acevedo-Arozena *et al.*, 2008). In-depth characterization of draggen mice, a novel mouse model of myotonia and periodic paralysis, found alterations in whole animal metabolism caused by *Scn4a* mutations accompanied by inappropriate AMPK activation in mice.

## Materials and methods

### Ethics statement

Consent for all neurophysiological tests was obtained, with tests performed as previously described (Fournier *et al.*, 2004, 2006; Tan *et al.*, 2011). All animal studies were carried out in accordance with UK Home Office legislation and local ethical guidelines. Mice characterization followed ARRIVE guidelines.

### Mice

Mice were kept under a controlled 12-h light cycle and had free access to water and were fed *ad libitum *on a commercial diet (SDS). Draggen mice were identified in the course of a dominant modifier ENU screen (Acevedo-Arozena *et al.*, 2008). Briefly, BALB/c male mice were intraperitoneally injected twice with 100 mg/kg ENU at 12 weeks of age and crossed to congenic C57BL/6J females. An individual draggen G1 founder showing intermittent hind-limb immobility or dragging attacks was initially identified and subsequently backcrossed onto C57BL/6J to assess inheritance. Affected individuals from the initial backcross showing hind-limb dragging attacks were used for a genome-wide scan that identified a critical region on mouse chromosome 11 that included *Scn4a*. The *Scn4a* gene was sequenced as a candidate gene and a missense mutation was subsequently identified by Sanger sequencing. Mice used in this study are at least N3 (three generations of backcross onto C57BL/6J); the colony is currently on N8. The draggen official allele name is: *Scn4a^m1Aaa^. Scn4a^M1592V^*^/^*^+^* [FVB.129S4(B6)-*Scn4a^tm1.1Ljh^*/J] mice were purchased from Jackson laboratories on a congenic FVB background and maintained by crossing to C57BL/6J for comparison with draggen mice. *Scn4a^M1592V^*^/^*^+^* mice used were backcrossed to C57BL/6J for at least two generations. All genotyping was performed by pyrosequencing (primer sequences available on request).

#### Phenotyping tests

For phenotyping tests, see the online Supplementary material.

### Dragging scoring

To quantify the age at onset and frequency of hind-limb immobility attacks in mice, we established a scoring system in which all mice were tested once a week from weaning until 60 weeks of age. It was observed that immobility events in draggen mice were more often seen on first opening of the cage in the morning. Thus, in the early morning, undisturbed mice were briefly held by the tail and then left to rear in their home cage. Hind-limb dragging was then recorded as present or absent by a trained technician.

### Electromyogram

EMGs were obtained from calf, hamstring, triceps and paraspinal muscles with concentric needle electrodes in isoflurane anaesthetized male mice at 30 weeks of age (*n = *5 per genotype). Young wild-type and non-affected males at 8–9 weeks of age (*n = *4 per genotype), and 60-week-old wild-type and non-hind-limb dragging females (*n = *4 per genotype) were also evaluated.

For chronic EMG, three draggen male mice were implanted with a wireless EMG system (Data Sciences F20-EET, Gold system). A transmitter was surgically implanted through an incision on the back, with leads from the transmitter leading, subcutaneously, to the right hind-limb. Two electrodes for the EMGs were attached to the gastrocnemius muscle. The surgeries were conducted with the mouse under ketamine–xylanine anaesthesia. A post-surgery period of 1 week was given to each mouse to ensure a full recovery. At the end of the recovery period, we recorded the EMG signal uninterruptedly. Sampling rate was 500 Hz and data were processed using SleepSign software (Kissei Comtec). The mice were singly housed and free to move in the cage. We continuously acquired videos with a computer synchronized with the EMG recordings. Videos were visually scored and immobility attacks were identified by at least two independent observers.

#### Physiological assessment of muscle force and *in vitro* electrophysiology analysis

For physiological assessment of muscle force and *in vitro* electrophysiology analysis, see the online Supplementary material.

### Histology

Young and adult male wild-type and draggen littermate (*n = *3) hind-limb muscles (tibialis anterior, extensor digitorum longus, gastrocnemius and soleus) were dissected at stated ages, weighed and snap frozen and 10-µm sections were cut in a cryostat. Tibialis anterior sections were stained using haematoxylin and eosin (and NADH-tetrazolium reductase) and an antibody-based stain for myosin heavy chain isoforms (IIa and IIb) as previously published (Bloemberg and Quadrilatero, 2012), with Alexa Fluor® 488 and 555 as secondary antibodies. Fibre numbers were recorded using ImageJ software (NIH). Fibre cross-sectional areas were measured for different fibre types (tibialis anterior, soleus) and fibre count and type percentages were assessed. For pups: post-natal Day 0 pups were sacrificed and formalin fixed. They were cut in longitudinal body halves and sections were stained with haematoxylin and eosin.

### Transmission electron microscopy

Mice were perfused with a mix of 2% glutaraldehyde and 2% paraformaldehyde in PBS. Hind-limb muscles were dissected and placed in 1% OsO4 on ice for 1 h followed by two 5-min washes with phosphate buffer (100 mM NaH_2_PO4/Na_2_HPO_4_.2H_2_0). The samples were dehydrated through an ethanol series (30%, 50%, 70%, 95% and absolute) and transferred to a 1:1 mixture of Epon/Araldite and acetone and incubated overnight with mixing. The solution was then replaced with a 1:1 mixture of 100% Epon/Araldite and incubated for 6 h, followed by another replacement with fresh Epon/Araldite and overnight incubation with mixing. Samples were transferred to fresh Epon/Araldite in embedding moulds and oriented as required. Polymerization of the Epon/Araldite mould was completed at 65°C for 48 h. Seventy nanometre sections were produced and stained with saturated uranyl acetate and Reynolds’ lead citrate. All images were obtained with a Tecnai 12 BioTWIN made by FEI.

### Immuno-blot analysis

Primary antibodies against p-AMPKα (Thr172) and total AMPKα were obtained from Cell Signalling. Tibialis anterior and extensor digitorum longus muscles from draggen, M1592V and age-matched wild-type littermate controls were homogenized in 10 volumes of RIPA buffer (with phosphatase and protease inhibitor cocktails from Roche). After spinning the homogenate at 12 000*g* at 4°C, the supernatant was used for western blot analysis using pre-cast SDS gels (Invitrogen). The Odyssey infrared imaging system (Li-Cor) was used for band detection and quantification.

### Statistical analysis

Data is presented as the mean ± standard error of the mean (SEM). Comparisons between two groups were made using the two-tailed Student’s *t*-test and Mann-Whitney. ANOVA with *post hoc* analysis (Bonferroni and Tukey) was used as appropriate. For survival and age-at-onset analysis, the Log-Rank test was performed. *P*-values were determined by SPSS v.19 software. *P* ≤ 0.05 was considered statistically significant.

## Results

### A novel mutation in *SCN4A* leads to myotonia and periodic paralysis

**Figure 1:**
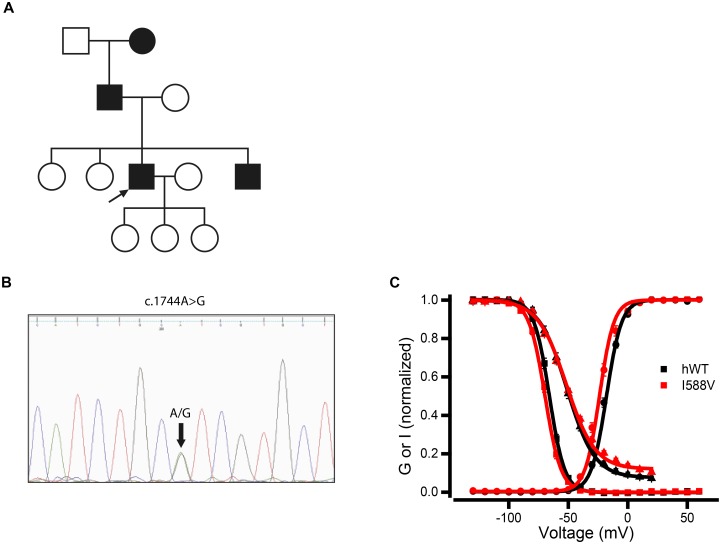
Mutations in *SCN4A* and *Scn4a* and functional characterization. (**A**) Family history of the c.1762A>G; I588V mutation. Proband indicated by arrow, affected family members are shaded. Females represented by circles and males by quadrangles. (**B**) Sequence trace showing the *Scn4a* c.1744A>G; p.I582V mutation in draggen mice. (**C**) Voltage dependence of activation (circles), fast inactivation (squares) and slow inactivation (triangles) for human (h) wild-type (black) and I588V (red) hNa_v_1.4 channels. The *y*-axis shows normalized conductance (activation) or current (fast and slow inactivation). The lines through the data are the best fit of the Boltzmann equation to the data. Data and statistical analysis are represented in Table 1.

A novel *SCN4A* mutation was identified in a patient presenting with myotonia and periodic paralysis. The mutation identified (c.1762A>G;p.I588V, *SCN4A^I588V^*) is located within the transmembrane S1 region of domain II. Mutations within this region have not been previously identified as causative of myotonia or periodic paralysis. The I588V mutation has not been documented in the exome variant server, 1000 Genomes, Leiden Open Variation Database or dbSNP. The 70-year-old male patient presented with a history of muscle stiffness and weakness from the age of eight. The stiffness was worst in his calves and worsened by exertion. The patient also noted episodes of weakness that were precipitated by running which would persist for weeks before complete resolution. In the past few years the episodes of weakness became more frequent, severe and prolonged, occurring once every 2–3 weeks. The episodes of stiffness have also persisted, primarily occurring following rest after exertion, with no reported sensitivity to cold. There is evidence of a family history (Fig. 1A), with a severely affected younger brother, father and paternal grandmother. The patient’s three daughters and two sisters are reported to be unaffected but were unavailable for assessment. Physical examination of the patient demonstrated myotonia of the eyelids that did not worsen with exertion. The patient had normal tone and power and reflexes throughout but some atrophy of the calves was noted. Neurophysiological testing demonstrated the presence of myotonia that worsened after the muscles were cooled (data not shown). Short exercise testing was normal both at room temperature and following cooling. In the long exercise test there was a decrease in amplitude of 38% and a concordant 58% of the area of the compound muscle action potential from baseline, which is abnormal (Tan *et al.*, 2011) (Supplementary Fig. 1A). The history of episodic weakness and stiffness from childhood alongside evidence of myotonia and a positive long exercise test was consistent with a clinical diagnosis of hyperkalaemic periodic paralysis together with paramyotonia congenita.

### Generation of a novel mouse model (draggen) carrying the equivalent point mutation in *Scn4a*

Through an unbiased large-scale ENU mutagenesis screen for dominant mutations (Nolan *et al.*, 2000), we identified a mutant mouse, named draggen (*Dgn*/*+*), with unprovoked intermittent attacks of hind-limb dragging or immobility. After positional cloning, a missense mutation in the mouse *Scn4a* gene was identified in the critical region of mouse chromosome 11, which segregated with the intermittent dragging phenotype. Once the mutation was identified, the colony was subsequently maintained by genotyping, with immobility attacks only affecting draggen mice. Remarkably, the mutation identified (c.1744A>G; p.I582V or *Scn4a^I582V^*) is the mouse equivalent to the newly identified human *SCN4A^I588V^* mutation described above (Fig. 1B). The mutated isoleucine residue is conserved between mice and humans and across species, and is also conserved in all mouse and human sodium voltage-gated channel paralogues (Supplementary Fig. 1B).

### *In vitro* electrophysiological characterization of the novel *SCN4A* and *Scn4a* mutations

To evaluate the electrophysiology of the novel *Scn4a^I582V^* and *SCN4A^I588V^* mutations, HEK293 cells were transfected with cDNA coding for the mouse or human wild-type or mutant Na_v_1.4 channel. Channel properties were analysed using whole-cell patch clamp. Voltage dependence of channel activation was shifted 6 mV in the hyperpolarizing direction in the mutant for both mouse and human clones (*P* < 0.005, *n = *10–25) (Fig. 1C, Supplementary Fig. 1C and Table 1), suggesting a gain of function. The voltage dependence of fast inactivation was shifted 3 mV in the hyperpolarizing direction for both mouse and human mutant channels [*P = *0.06 for mouse clone (*n = *13–15), *P < *0.01 for human clone (*n = *18–20)] (Fig. 1C, Supplementary Fig. 1C and Table 1). No significant differences were detected in the voltage dependence of slow inactivation (Fig. 1C, Supplementary Fig. 1C and Table 1).

**Table 1:** Electrophysiological properties of wild-type and mutant Na_v_1.4 channels

	Activation		Fast inactivation		Slow inactivation		
Clone	*n*	V1/2 (mV)	*n*	V1/2 (mV)	*n*	V1/2 (mV)	C (%)
hWT	25	−18.8 ± 0.7	20	−65.9 ± 0.7	15	−52.8 ± 1.4	9.8 ± 1.1
I588V	21	−24.8 ± 0.6***	18	−68.9 ± 0.8**	15	−53.7 ± 1.2	13.2 ± 0.8
mWT	12	−17.5 ± 0.6	15	−65.1 ± 1.2	10	−49.6 ± 1.2	7.8 ± 1.6
Draggen	10	−23.1 ± 1.1***	13	−68.5 ± 1.2	5	−52.5 ± 3.4	10.5 ± 3.0

Voltage values at which half of the channels (V1/2) were activated (Activation) or inactivated (fast and slow inactivation) and the offset current (C) of slow inactivation are stated for human and mouse wild-type (hWT and mWT), human I588V and Draggen mouse (I582V) mutant channels. Statistical comparisons are between the wild-type and mutant clones. **P < 0.01, ***P < 0.001.

### Myotonia and hind-limb immobility attacks in draggen mice

**Figure 2:**
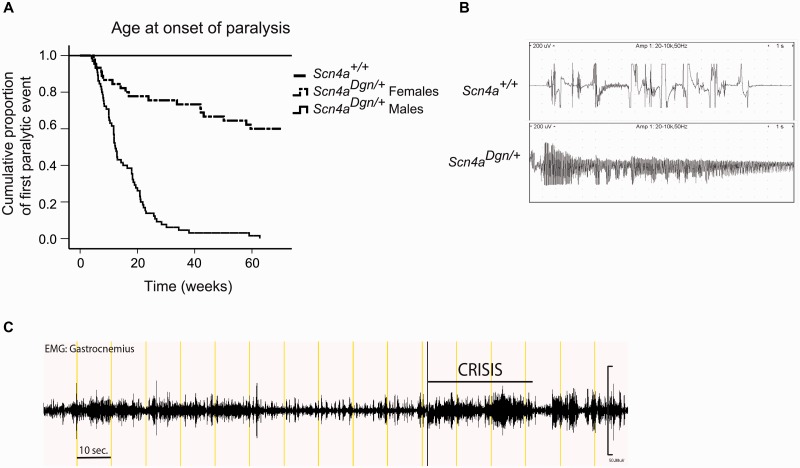
Draggen mice suffer from immobility attacks and myotonia. (**A**) Age at onset of hind-limb immobility attacks of draggen males and females up to 60 weeks of age. Males: *n = *65, females: *n = *46. Log-Rank comparison between overall males and females: *P < *0.001. All 65 males and 17 (38%) draggen females had at least one hind-limb dragging episode. Differences in age at onset between males and females were detected when selecting dragging-affected females (*P = *0.013). (**B**). Electromyography traces representative of male wild-type and draggen hind-limb muscles. Wild-type recording illustrates artefacts from multiple needle movements that failed to evoke myotonic discharges in all wild-type mice whereas long myotonic runs were always elicited in draggen mice (*n = *5 mice per group). (**C**) Chronic EMG recordings from the gastrocnemius muscle of a draggen male mouse before, during and after an intermittent immobility event (named crisis). High EMG activity accompanies the hind-limb dragging episode (highlighted as ‘crisis’).

Draggen mice (*Scn4a^Dgn^*^/^*^+^)* suffered from unprovoked intermittent hind-limb immobility attacks when the mouse was not able to move the hind-limbs. This was followed by a total recovery in mobility after a short period of time usually lasting a few seconds (Supplementary Video 1). To systematically assess the hind-limb dragging onset and frequency, mutant mice were subjected to a test once a week from 3 weeks of age; hind-limb immobility attacks were scored as present or absent. Through this process, a clear difference in the penetrance of the observed intermittent dragging phenotype was noted between male and female mutant mice (Fig. 2A, *P < *0.001). All males carrying the draggen mutation showed at least one hind-limb immobility episode by 60 weeks of age (100% penetrance), with an average age at onset of ∼16 weeks of age. However, only 38% of draggen females displayed an episode at least once during the same period, with an average age at onset of ∼25 weeks of age, which is significantly different from that observed in males (*P = *0.013). For those mice showing the intermittent attacks, there is a large variation in the age at which the first attack is detected, with extreme cases showing the first episode as early as weaning (3 weeks of age) or as late as 50–60 weeks of age. In addition, the frequency of hind-limb dragging attacks that was observed over the lifetime of individual mice was also different between males (average 8 ± 0.9 episodes up to 60-weeks of age) and affected females (2.6 ± 0.4 episodes; *P < *0.001).

As the proband presented with myotonia, draggen mice were subjected to electromyography (EMG) of hind-limb muscles. Mutant mice showed striking myotonic runs after light movement of the needle that were not present in wild-type littermate controls (Fig. 2B). Interestingly, myotonia was present in all of the draggen mice assessed. These included young males tested before their first recorded dragging attack, as well as aged draggen females that never had any recorded hind-limb immobility event (data not shown). Hence, myotonia is fully penetrant in draggen mutants, including females that have never shown a dragging episode based on our scoring system.

To study the muscle activity during the hind-limb immobility attacks, we performed chronic, wireless EMG on freely moving singly housed draggen males. We synchronized the EMG recordings with video monitoring to focus on the EMG activity during the dragging events. The intermittent immobility attacks were accompanied by high EMG activity, which continued throughout the whole event (Fig. 2C and Supplementary Fig. 2). Overall, this intense EMG activity suggests that the immobility or dragging attacks are not produced by muscle weakness in draggen mice.

### The draggen mutation is homozygous lethal

Heterozygous draggen mice (*Scn4a^Dgn^*^/^*^+^*) had a normal lifespan up to 2 years of age (Supplementary Fig. 3A). However, although homozygous draggen mice (*Scn4a^Dgn^*^/^*^Dgn^*) are born at Mendelian ratios from heterozygous intercross matings (21 *Scn4a^Dgn^*^/^*^Dgn^* from a total of 90 pups born), we did not observe any homozygous pups older than 2 days. *Scn4a^Dgn^*^/^*^Dgn^* mice died perinatally with noted breathing difficulties, confirmed by the observation of collapsed lungs (Supplementary Fig. 3B), but otherwise morphologically normal organs. Notably, skeletal muscle, including diaphragm, was also morphologically normal (data not shown), suggesting that the cause of death may be due to a functional impairment of muscles required for breathing and suckling.

To confirm that the homozygous lethality was due to the *Scn4a* draggen mutation, an allelic complementation cross was performed with a previously generated *Scn4a* knock-in mouse strain (*Scn4a^M1592V^*^/^*^+^*) (Hayward *et al.*, 2008). *Scn4a^Dgn^*^/^*^M1592V^* compound mutant mice were born at Mendelian ratios (15 *Scn4a^Dgn^*^/^*^M1592V^* out of 59 pups born) but also died perinatally with similar difficulties in breathing and lung morphology (Supplementary Fig. 3C). We therefore concluded that the lethality was due to *Scn4a* allelic non-complementation.

### Reduced muscle force generated by draggen muscles

**Figure 3:**
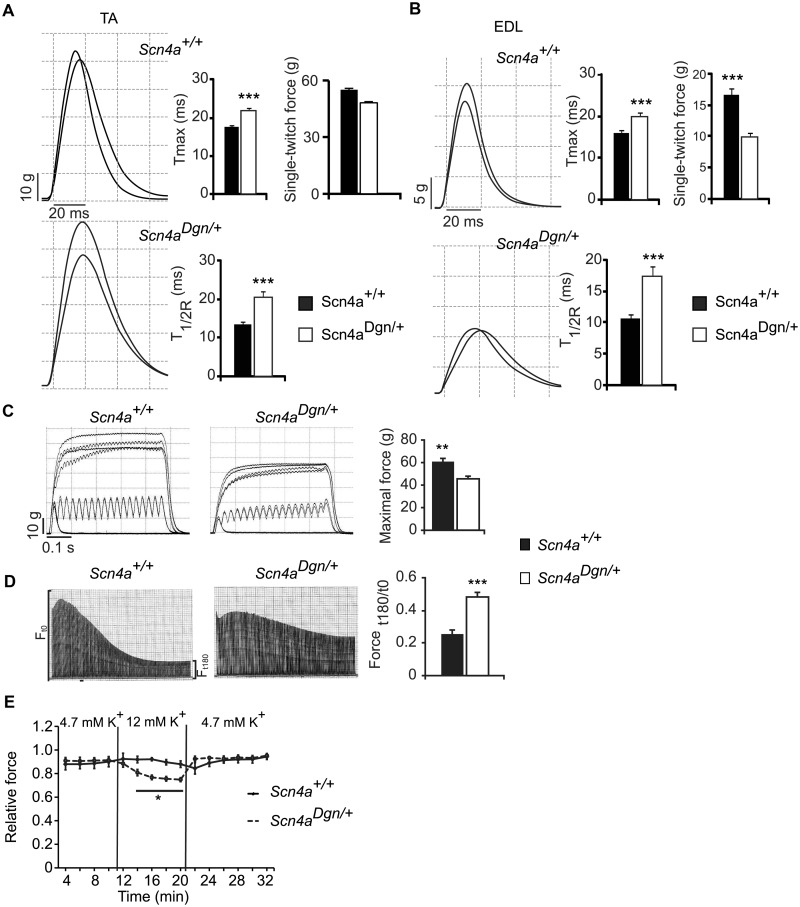
Draggen mice have muscle weakness. *In vivo* physiological assessment of hind-limb muscles of 60-week-old male mice. *Scn4a^+^*^/^*^+^* (*n = *12); *Scn4a^Dgn^*^/^*^+^* (*n = *14). (**A**) Tibialis anterior (TA) muscle force showed that the time to peak force (T_max_) and half-time relaxation time (T_1/2R_) were significantly longer in draggen mice than wild-type controls. For T_max_ (*Scn4a^+^*^/^*^+^* = 17.5 ms; *Scn4a^+^*^/^*^+^*; *Scn4a^Dgn^*^/^*^+^* = 21.9 ms; *P < *0.001). For T_1/2R_ (*Scn4a^+^*^/^*^+^* = 13.3 ms; *Scn4a^Dgn^*^/^*^+^* = 20.6 ms; *P < *0.001). Single twitch force for tibialis anterior muscles was not significantly different between wild-type and draggen muscles (*Scn4a^+^*^/^*^+^* = 54.1 g; *Scn4a^Dgn^*^/^*^+^* = 45.7 g; *P = *0.19). The two traces shown per image represent the right and left hind-limb from the same animal. (**B**) Extensor digitorum longus (EDL) muscles in draggen mice also took longer to reach both T_max_ and T_1/2R_ than wild-type (T_max_: *Scn4a^+^*^/^*^+^ = *15.9 ms; *Scn4a^Dgn^*^/^*^+^ = *20.0 ms; *P < *0.001. T_1/2R_: *Scn4a^+^*^/^*^+^ = *10.7 ms; *Scn4a^Dgn^*^/^*^+^ = *17.3 ms; *P < *0.001). Single twitch force was also determined for extensor digitorum longus muscles, with wild-type muscles exerting more force than draggen muscles (*Scn4a^+^*^/^*^+^ = *16.7 g; *Scn4a^Dgn^*^/^*^+^ = *9.8 g; *P < *0.001). (**C**) Extensor digitorum longus tetanic force generated by draggen mice (45.8) is reduced compared to wild-type littermates (60.5) (*P = *0.003). (**D**) Representative traces of tetanic tension from wild-type and draggen extensor digitorum longus muscles. The fatigue index (FI) is increased for draggen muscle (0.48) when compared to wild-type (0.25) (*P < *0.001). (**E**) High potassium levels diminish force generated by extensor digitorum longus muscles of draggen mice *ex vivo*. *Scn4a^+^*^/^*^+^* (*n = *7); *Scn4a^Dgn^*^/^*^+^* (*n = *9) (*P*-values: 14 min: *P = *0.017; 16–20 min: *P < *0.003). The force generated by extensor digitorum longus was measured every 2 min with muscles submerged in a bath with normal (4.75 mM) and high (12 mM) potassium concentrations for 10 min in each condition. Data are expressed as mean ± SEM. *P < 0.05; **P < 0.01; ***P < 0.001.

Muscle force, measured by grip-strength (Supplementary Fig. 4A), and motor coordination, measured as locotronic performance (Supplementary Fig. 4B), progressively deteriorated in draggen mice. To further assess muscle force *in vivo*, the physiology of the hind-limb tibialis anterioris and extensor digitorum longus muscles were studied in a cohort of wild-type and draggen male littermates at 60 weeks of age. For both the tibialis anterior and extensor digitorum longus muscles, the time at which maximum contraction (T_max_) and half relaxation (T_1/2R_) were achieved was significantly higher in draggen mice compared to wild-type littermates (Fig. 3A and B, *P < *0.001 for all comparisons). Moreover, the single twitch force (Fig. 3B) and the maximum force generated by the extensor digitorum longus muscles (Fig. 3C) were lower in draggen mice when compared with wild-type (*P < *0.001 for all comparisons). This was not due to a difference in tibialis anterior and extensor digitorum longus muscles weight (data not shown). Fast-twitch muscles, such as the extensor digitorum longus, normally fatigue rapidly and cannot maintain force when repeatedly stimulated, whereas slow-twitch muscles are more resistant to fatigue. Typical fatigue traces show that the extensor digitorum longus of draggen mice becomes more fatigue-resistant after stimulation (Fig. 3D). The fatigue index of draggen extensor digitorum longus muscles was almost double that of the wild-type littermate controls (*P < *0.001). This is usually observed in slow-twitch muscles and muscles adapted to repeated contractile activity or endurance training, suggesting potential metabolic changes in draggen muscle. Motor units were also estimated in the extensor digitorum longus muscles of wild-type and draggen mice and no significant differences were observed (*P = *0.6, data not shown), suggesting that the muscle weakness and fatigue characteristics in draggen mice are not caused by denervation or axon/motor unit dysfunction.

### High extracellular potassium levels can elicit muscle weakness in draggen muscle

Periodic paralysis is characterized by attacks of muscle weakness, which can be elicited in some patients by changes in extracellular potassium levels (Moxley *et al.*, 1989; Vicart *et al.*, 2004; Hayward *et al.*, 2008). The effect of different potassium levels in muscle force was therefore measured *ex vivo* in the extensor digitorum longus from 60-week-old wild-type and draggen male mice in the presence of low (2 mM), normal (4.75 mM) and high (12 mM) potassium levels. Wild-type extensor digitorum longus muscles exerted similar maximal force in all conditions. In contrast, in draggen extensor digitorum longus muscles high, but not low (data not shown), potassium levels resulted in a significant force reduction (Fig. 3E). Hence, a degree of muscle weakness can be elicited *ex vivo* by high potassium in draggen muscle.

### Progressive myopathy in draggen mice

**Figure 4:**
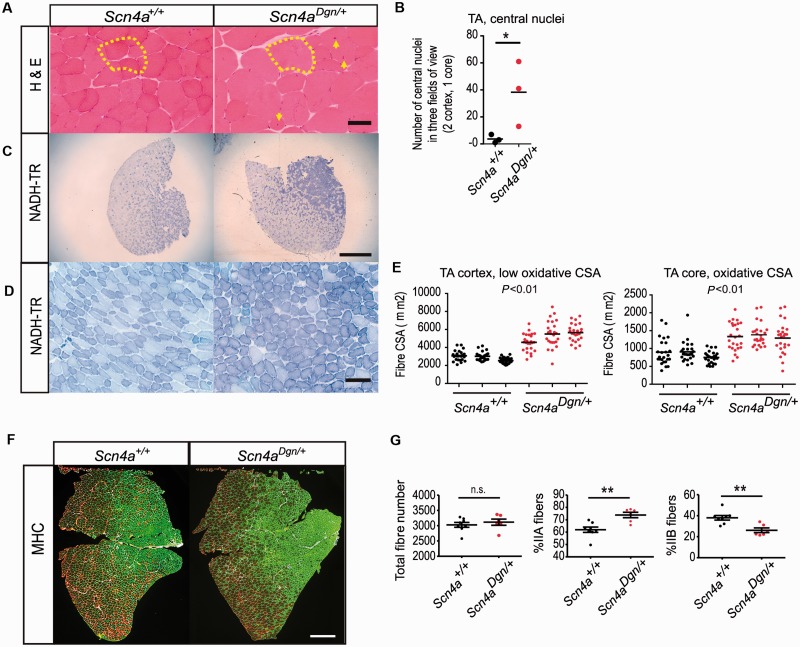
Progressive myopathy in draggen mice. (**A**) Pathological assessment of tibialis anterior (TA) muscle of 60-week-old male wild-type and draggen littermates. Haematoxylin and eosin (H&E) of the tibialis anterior cortex shows central nuclei and hypertrophic muscle fibres in draggen muscle (delimited in yellow area to show the size of one draggen fibre for comparison). Yellow arrows point to central nuclei. (**B**) Quantification of the central nuclei in the muscle fibres of wild-type and draggen tibialis anterior muscles (*n = *3 mice per group, *n = *3 fields per mouse; *P = *0.05). (**C**) NADH-TR activity staining shows more oxidative fibre grouping in the core of the draggen tibialis anterior muscles. (**D**) Higher magnification of the core of the tibialis anterior muscle stained with NADH-TH showing enlarged high oxidative fibre grouping in the draggen mice compare to the wild-type. (**E**) Quantification of fibre size (CSA, cross section area) in the tibialis anterior core and cortex showing enlarged high and low oxidative draggen fibres when compared to wild-type (*n = *3 per group, *P < *0.001 for both comparisons). (**F**) Representative MHC staining showing the fibre type in the tibialis anterior muscle of 12-week-old mice wild-type and draggen littermates (IIa fibres in green and IIb fibres in red). (**G**) Quantification of the total number and the percentage of IIa and IIb fibres. (*Scn4a^+^*^/^*^+^ n = *8; *Scn4a^Dgn^*^/^*^+^ n = *6 mice, *P = *0.002 for both comparisons). Data are expressed as mean ± SEM. *P < 0.05; **P < 0.01. Scale bars: **A** = 50 µm; C and **F** = 500 µm; **D** = 100 µm.

Some forms of periodic paralysis and myotonia have been associated with muscle histopathological alterations as patients age (Bradley *et al.*, 1990; Plassart *et al.*, 1994; Nagamitsu *et al.*, 2000; Schoser *et al.*, 2007). Tibialis anterior, extensor digitorum longus and soleus hind-limb muscles were therefore examined in male mice at two stages: young (12 weeks of age) and aged (60 weeks of age). At 60 weeks of age, haematoxylin and eosin staining revealed an increased number of central nuclei and the presence of larger muscle fibres in aged draggen tibialis anterior muscles compared to wild-type (Fig. 4A and B), providing evidence of a progressive damage/regeneration process. Interestingly, the soleus muscle was unaffected (data not shown). NADH-tetrazolium reductase (NADH-TR) staining of the tibialis anterior muscle at 60 weeks of age showed a fibre switch towards a more oxidative type together with fibre grouping, particularly in the more oxidative tibialis anterior core (Fig. 4C and D). Both the low oxidative fibres in the cortex and the highly oxidative fibres in the tibialis anterior core were significantly enlarged in aged draggen mice (Fig. 4E). Young (12 weeks of age) draggen tibialis anterior muscles did not have central nuclei (data not shown). Muscle myosin heavy chain (MHC) immunostaining was performed on young tibialis anterior muscle to quantify fibre types. Interestingly, at this stage there is already a significant degree of fibre switching towards a more oxidative type in draggen tibialis anterior muscle, in both males and females, regardless of whether they have presented with dragging attacks (Fig. 4F and G).

**Figure 5:**
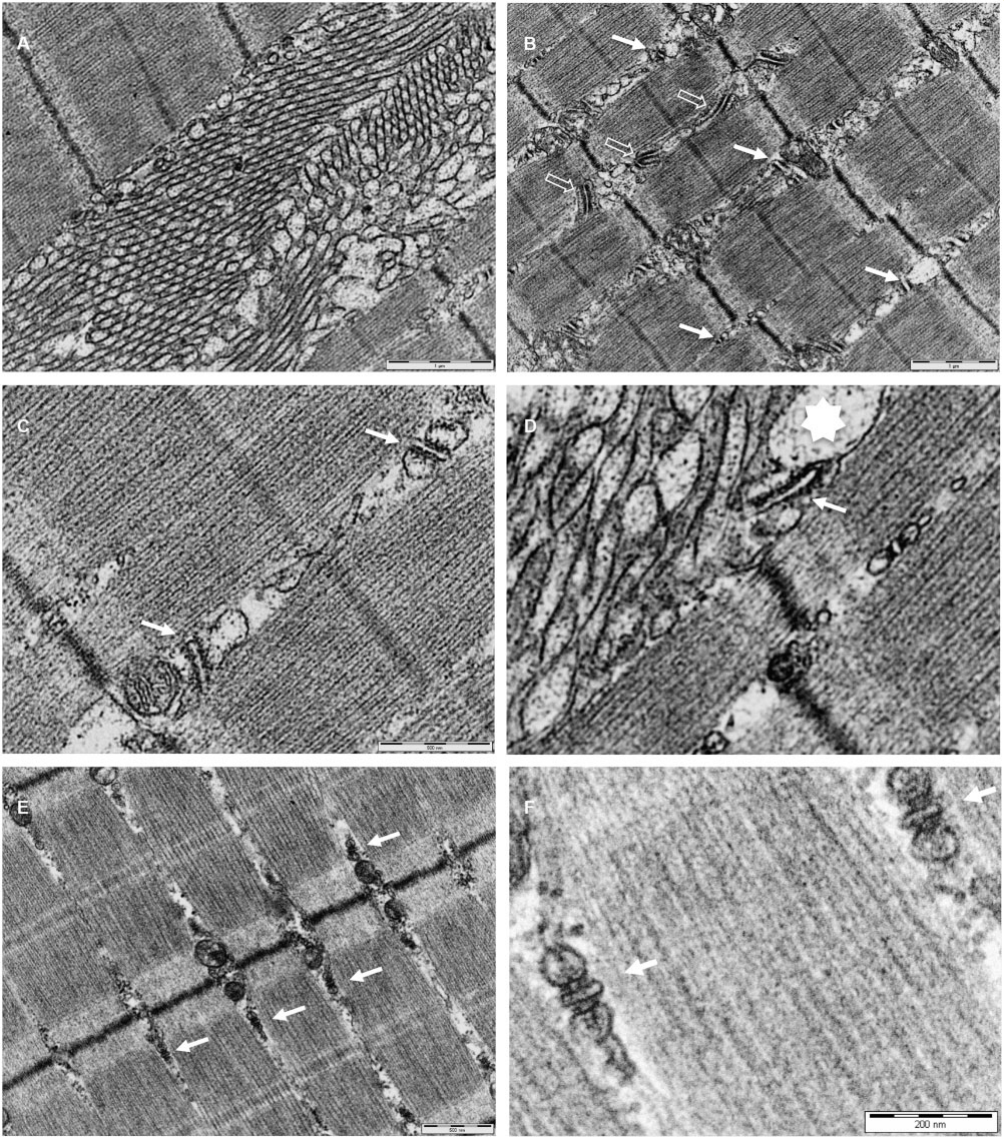
Tubular aggregates and vacuoles in skeletal muscle of draggen mice. Transmission electron microscopy analysis of the tibialis anterior muscle from draggen (**A–D**) and control mice (**E** and **F**). (**A**) Representative view of single-walled tubular aggregates that have accumulated between myofibrils. (**B**) Longitudinal section showing triads of different morphological appearances (filled arrows). Note the presence of densities between adjacent membranes (open arrows). (**C**) Detailed view of T tubules and sarcoplasmic reticulum of triads (filled arrows) of irregular size and orientation. (**D**) Detailed view of a tubular aggregate region showing the continuity of the sarcoplasmic reticulum from a presumed irregular triad (filled arrow) with a large vesicle (star). (**E** and **F**) Wide and detailed view, respectively, of triads of normal size and orientation (arrows) from a control sample.

Transmission electron microscopy analysis of the tibialis anterior muscle of two aged draggen and control male mice at ∼1.5 years of age was carried out. Representative observations from two draggen mice are shown in Fig. 5. The presence of tubular aggregates (Fig. 5A) as well as irregular triads (Fig. 5B and C) was revealed in ultrathin longitudinal sections (70 nm). Triads of irregular size and orientation were widespread in many fibres that did not show tubular aggregates. There is some evidence of continuity of the terminal cisternae of the triads with the large vesicles found in the proximity of tubular aggregates (Fig. 5D), in agreement with previous observations showing that tubular aggregates may originate from the sarcoplasmic reticulum (Engel *et al.*, 1970; Schiaffino, 2012). None of these hallmarks were detected in the age and sex-matched control sample (Fig. 5E and F). Thus, draggen mice show progressive muscle pathology, mimicking the muscle pathology seen in some myotonia and patients with periodic paralysis (Bradley *et al.*, 1990; Tengan *et al.*, 1994; Feng *et al.*, 2009, 2011; Luan *et al.*, 2009; Trip *et al.*, 2009).

### Metabolic abnormalities in draggen mice

**Figure 6:**
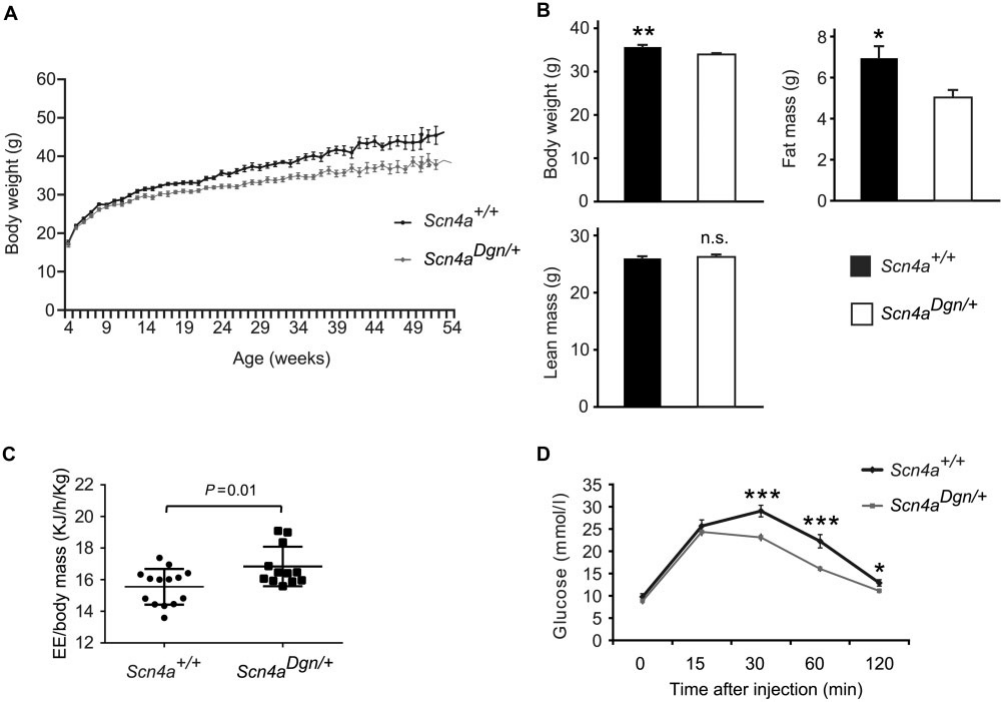
Draggen mutation affects body weight and metabolism. (**A**) Body weight (g) of male wild-type and draggen mice from 5 to 50 weeks of age. Draggen mice gain less weight than their littermate controls (*P < *0.01 from 12 weeks; *P < *0.001 from 18 weeks onwards). At least 15 mice per genotype and time point were examined**.** (**B**) Body composition at 20 weeks of age in male mice. Draggen mice have less total body weight [*Scn4a^+^*^/^*^+^* (*n = *24); *Scn4a^Dgn^*^/^*^+^* (*n = *17), *P = *0.006] due to a reduction in total fat mass (*P = *0.02), with no changes in lean mass (*P = *0.65). (**C**) Whole body energy expenditure (KJ/h/kg) corrected by body weight (kg) shows draggen mice exerting higher whole body energy expenditure in the dark period (*P = *0.01). *Scn4a^+^*^/^*^+^* (*n = *14); *Scn4a^Dgn^*^/^*^+^* (*n = *12). (**D**) Glucose tolerance test curves during a 120-min intraperitoneal glucose injection (IPGTT) in 18-week-old male mice (*n = *15 per group; overall *P < *0.001; per individual time points: *P < *0.001 at T30 and T60, and *P = *0.02 at final time point T120). Data are expressed as mean ± SEM. *P < 0.05; **P < 0.01; ***P < 0.001; n.s. = not significant.

Male draggen mice, but not females, showed significantly reduced weight gain from 12 weeks of age compared to wild-type littermates (Fig. 6A and Supplementary Fig. 5A). The weight differences in males were not due to differences in body size, measured via femoral bone X-ray, or in food consumption, measured on a 24-h cycle (data not shown). Importantly, similar results were obtained when comparing the weights of wild-type and *Scn4a^M1592V^*^/^*^+^* male mice (Supplementary Fig. 5B). To further dissect differences in body composition, the amount of fat and lean mass was measured using Echo-MRI at 18 weeks of age. Draggen male mice displayed less total fat mass than their wild-type littermates (Fig. 6B; *P = *0.015). As draggen male mice have less fat mass yet consume similar amounts of food as wild-type littermates, their whole body energy expenditure was measured. Draggen mice had significantly higher whole body energy expenditure than wild-type littermates when we controlled for total body weight in the dark period of a 24 h cycle (Fig. 6C, *P = *0.01). Weight differences might also be explained by differences in circulating leptin levels (Oswal and Yeo, 2010). We found a trend towards a reduction in serum leptin levels in draggen mice compared to wild-type at 40 weeks of age (Supplementary Fig. 5C, *P = *0.07). We also tested glucose metabolism and found that draggen mice re-established plasma glucose levels more efficiently than wild-type littermates following a glucose tolerance test (Fig. 6D), despite similar baseline insulin levels (Supplementary Fig. 5D). In summary, mutations in the muscle specific *Scn4a* gene have profound effects on the general metabolic status of the whole animal.

### AMPK is constitutively activated in draggen and *Scn4a^M1592V^*^/^*^+^* skeletal muscle in mice with hind-limb immobility

**Figure 7:**
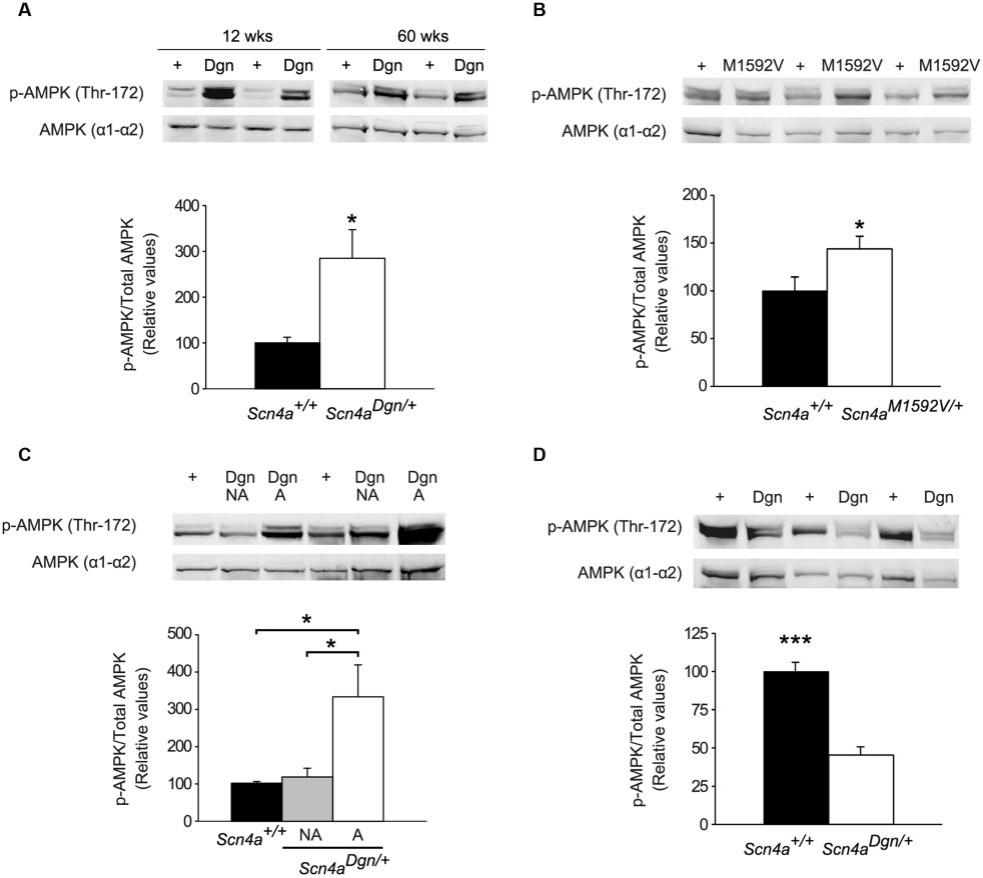
Changes in AMPK activation levels in mutant *Scn4a* hind-limb muscle. Immuno-blot analysis of mouse tibialis anterior muscle lysates using antibodies to activated AMPK [Phospho-AMPKα (Thr172) and total AMPK levels (α1–α2)]. AMPK activation is expressed as the coefficient between the p-AMPK/total-AMPK band intensities; graph values are relative to the average of each wild-type group. Protein loading was controlled by GAPDH (data not shown). (**A**) Representative images of immunoblots from young (12-week-old) and aged (60-week-old) wild-type or draggen mutant mice. In draggen mice the level of activated AMPK is higher than wild-type (*n = *6 per genotype; *P = *0.02). (**B**) Representative images of immunoblots from tibialis anterior muscle of wild-type and *Scn4a^M1592V^*^/^*^+^* mice, showing higher basal AMPK activation in *Scn4a^M1592V^*^/^*^+^* (*n = *4) when compared to wild-type (*n = *3); *P = *0.04. (**C**) Representative images of immunoblots from wild-type and draggen littermates that have not shown a hind-limb dragging attack (NA) and draggen mice that have already shown at least one immobility episode (A). AMPK activation is elevated only in draggen mice that have already shown immobility attacks (*n = *4 per group; wild-type versus NA, *P = *0.4; wild-type versus A, *P = *0.03; A versus NA, *P = *0.04). (**D**) Representative immunoblots of tetanically stimulated tibialis anterior and extensor digitorum longus muscles lysates from 60-week-old wild-type and draggen littermates mice induced after *in vivo* physiology recordings (including muscle force, fatigue characteristics and estimation of motor units). The quantification graph shows blunted AMPK activation levels in draggen muscles after stimulation when compared to wild-type (*n = *6 in both groups; *P < *0.001). Data are expressed as mean ± SEM. *P < 0.05; ***P < 0.001.

As muscle is a major contributor to the whole body energy expenditure and metabolic rate, we speculated that the systemic metabolic alterations in draggen mice may be a consequence of disturbed muscle energy demand that is required to re-establish ion balance in myotonic muscle or after intermittent immobility attacks. Muscle contractions increase the rate of ATP hydrolysis which, via increases in the concentrations of AMP and ADP, activates the key intracellular energy sensor AMPK (Winder and Hardie, 1996; Friedrichsen *et al.*, 2013), mainly via phosphorylation of Thr172 (Hawley *et al.*, 1996; Mu *et al.*, 2001). We therefore hypothesized that the metabolic alterations seen in draggen male mice may be mediated by differential AMPK activation in mutant muscles. To estimate AMPK activation, Thr172 phosphorylation was measured from tibialis anterior muscle in wild-type and draggen male mice at two ages: young (12 weeks) and aged (60 weeks). Total AMPK levels were similar between all genotypes at both ages. However, when compared to wild-type littermates, AMPK Thr172 phosphorylation was higher in both young and aged tibialis anterior muscles of draggen mice that have shown at least one immobility episode (Fig. 7A). Moreover, AMPK activation levels were also higher in the tibialis anterior of the *Scn4a^M1592V^*^/^*^+^* mice (Fig. 7B), which also showed intermittent immobility attacks using our scoring system. Surprisingly, this steady-state activation of AMPK is not observed in draggen mice that have not shown a hind-limb immobility episode using our scoring system (Fig. 7C), despite all tested draggen mice having underlying myotonia. Thus, higher activation levels of AMPK may be a general feature of mouse models carrying *Scn4a* mutations presenting with intermittent immobility attacks.

The higher basal levels of activated AMPK in draggen muscle suggest a possible functional link between AMPK activation levels and Na_v_1.4 channel physiology. We therefore investigated if AMPK activation could directly modify the electrophysiology of Na_v_1.4 channels *in vitro*. HEK293 cells transfected with Na_v_1.4 variants were incubated with the AMPK activator AICAR or cotransfected with a constitutively active truncated AMPK clone AMPKα1(1–312). In these conditions, changes in AMPK activation levels did not have any effects on voltage dependence of wild-type or mutant Na_v_1.4 channel activation or fast inactivation (Supplementary Fig. 6). Thus, at least *in vitro*, AMPK activation is not sufficient to significantly alter the electrophysiology of the Na_v_1.4 channel.

### Blunted AMPK activation in draggen muscles after *in vivo* stimulation

In patients, attacks of periodic paralysis can occur after physical exertion, implying a potential link between energy demand and paralysis episodes. To mimic exercise in the mice, we tetanically stimulated the tibialis anterior and extensor digitorum longus muscles of mice that have been previously used for the above *in vivo* physiology recordings and measured AMPK activation levels. Remarkably, muscles from draggen mice that had previously shown immobility episodes were not able to activate AMPK to the same degree as wild-type littermate controls after the tetanic stimulation (Fig. 7D). Thus, AMPK activation is perturbed, both in basal conditions and after stimulation in draggen muscles.

## Discussion

Here we report a novel pathological mutation in the *SCN4A* gene in a patient presenting with myotonia and periodic paralysis. We have also produced its mouse model, draggen, carrying the equivalent point mutation in the mouse *Scn4a* gene. All tested draggen mice had myotonia, and all draggen males and a subset of draggen females also developed spontaneous hind-limb immobility attacks. This is underlined by a progressive myopathy and muscle fibre switching towards a more oxidative type. Interestingly, male draggen mice have systemic metabolic abnormalities, including diminished fat mass and improved glucose tolerance. This is accompanied by changes in AMPK activation, both in basal conditions and after muscle stimulation, in mice that present with dragging attacks.

The majority of dominant *SCN4A* causative mutations in periodic paralysis with myotonia have been found in the pore forming segments S5–S6 or the voltage sensor segment S4 of Na_v_1.4. The mutation identified here is located in the transmembrane segment S1 of domain II, where no other *SCN4A* mutations have been previously described. There are other mutations in the homologous S1 segment of domain IV of the Na_v_1.4 channel (Wagner *et al.*, 1997) presenting features of hyperkalaemic periodic paralysis and paramyotonia congenita with reported gender differences. There are also reported mutations in the homologous S1 residue of domain I of the Na_v_1.4 channel (Petitprez *et al.*, 2008), as well as in another sodium voltage-gated paralogue (Na_v_1.7) (Cheng *et al.*, 2008) that cause similar effects to I588V, causing a hyperpolarizing shift in the voltage dependence of activation. The hyperpolarizing shift in the voltage dependence of activation found in I588V Na_v_1.4 channels is found for several mutations associated with myotonia (Petitprez *et al.*, 2008; Kokunai *et al.*, 2012; Yoshinaga *et al.*, 2012), and would lower the activation channel threshold and contribute towards myotonia. Depolarization caused by increased sodium channel activity and the small enhancement of fast inactivation may contribute towards reduced Na_v_1.4 channel availability and muscle weakness. Many hyperkalaemic periodic paralysis mutations display attenuated slow inactivation whereas the slow inactivation of I588V mutant channel was wild-type like. However, some hyperkalaemic periodic paralysis mutations present with intact or increased slow inactivation (Hayward *et al.*, 1999; Bendahhou *et al.*, 2000), suggesting that Na_v_1.4 mutations can lead to hyperkalaemic periodic paralysis without disruption of slow inactivation.

We were able to elicit muscle weakness *ex vivo* in draggen muscles by increasing the extracellular potassium concentration. However, although highly statistically significant, the drop in muscle force (∼25%) in draggen muscle after increasing [K^+^] was less pronounced than the drop previously published in *Scn4a^M1592V^*^/^*^+^* muscles; a drop in muscle force of ∼70% that was confirmed with our experimental set-up (data not shown). This may correlate with the fact that *in vitro* these novel human and draggen mutations had only small effects on voltage dependence channel inactivation. Indeed, the intermittent attacks seen in draggen mice are accompanied by high EMG activity, suggesting that they are not produced by muscle weakness. Nevertheless, in the patient described here, the I588V *SCN4A* mutation unequivocally leads to myotonia and periodic paralysis.

Importantly, we report a progressive myopathy in draggen mice, with the appearance of tubular aggregates, vacuoles and associated muscle atrophy. Thus, it is not surprising to find reduced muscle force in aged draggen mice. Although we did not see any tubular aggregates in our control muscles, it should be noted that tubular aggregates have been previously reported in aged inbred mice and could be a consequence of the ageing process (Agbulut *et al.*, 2000). However, similar pathological features have been described in cases of periodic paralysis and myotonia (Bradley *et al.*, 1990; Schoser *et al.*, 2007; Luan *et al.*, 2009; Feng *et al.*, 2011). Therefore, draggen mice offer a novel excellent model to study the role of tubular aggregates and vacuoles in the pathophysiology of S*CN4A* channelopathies.

During the course of this investigation, we analysed *Scn4a^M1592V^*^/^*^+^* mice that have previously been characterized but were not reported to develop intermittent hind-limb dragging attacks (Hayward *et al.*, 2008). Using our scoring system, we were able to detect hind-limb immobility attacks on *Scn4a^M1592V^*^/^*^+^* mice that were indistinguishable from those seen in draggen mice (data not shown). The previous absence of reported immobility attacks in *Scn4a^M1592V^*^/^*^+^* may be due to differences in the genetic background between the strain used here and that used in the initial characterization of the *Scn4a^M1592V^*^/^*^+^* mice (Hayward *et al.*, 2008).

The draggen mutation causes EMG myotonia in all tested draggen males and females, indicating full penetrance of this phenotype. However, this is not the case for the intermittent immobility events; the mutation affects all males, but only a subset of mutant females. These sex differences are not explained by differences in *Scn4a* allelic expression levels between males and females (data not shown). This suggests that modifying factors differing between males and females, other than the mutation itself, may have an impact on the biophysics of the mutant Na_v_1.4 channel, making males more prone to suffer from immobility attacks. Sex differences in phenotypic penetrance have been previously reported in forms of periodic paralysis (Wagner *et al.*, 1997; Kim *et al.*, 2004; Li *et al.*, 2012; Ke *et al.*, 2013) and in a mouse model of hypokalaemic periodic paralysis (Wu *et al.*, 2011). The identification of these potential sex-specific modulating factors could make them attractive targets for therapeutic intervention.

To investigate possible factors that might modify the properties of the mutant Na_v_1.4 channel, we looked into the whole body metabolism of draggen mice. Lower body weight in draggen males was accompanied by general metabolic alterations, including enhanced whole body energy expenditure and glucose tolerance, which together potentially contribute to the leaner phenotype. Importantly, we also observed weight differences between *Scn4a^M1592V^*^/^*^+^* and wild-type males. Overall, these data show that mutations in the muscle specific *Scn4a* gene can produce systemic metabolic alterations.

We hypothesized that the leaner phenotype seen in draggen males could reflect differences in energy homeostasis. Indeed, basal AMPK activation levels are higher in draggen muscles that have shown at least one episode of hind-limb dragging. This could potentially explain the glucose tolerance (Friedrichsen *et al.*, 2013) as well as the higher energy expenditure phenotypes in draggen males. However, AMPK activation was not significantly increased in draggen muscle before showing at least an immobility episode reported using our scoring system. This suggests that myotonia alone may not be sufficient to drive the increase in steady-state AMPK activation levels. However, the severity of myotonia may vary between individual draggen mice; in this scenario, mice with the more severe myotonia could have more involuntary contractile activity, presented as episodes of immobility, leading to a depletion of energy and a subsequent activation of AMPK. This, in turn, could result in endurance training-like adaptations such as blunted AMPK activation after stimulation (Mortensen *et al.*, 2013). Interestingly, baseline AMPK activation levels were also higher in *Scn4a^M1592V^*^/^*^+^* muscles, confirming that the rise in AMPK activation levels is not unique to draggen muscles and could be a generalized feature of *Scn4a* mouse models showing myotonia and hind-limb dragging attacks.

AMPK activation has been shown to affect the properties of ion channels expressed in skeletal muscle such as Kir2.1 and K_ATP_ channels (Alesutan *et al.*, 2011; Yoshida *et al.*, 2012), and might couple changes in metabolism to cell excitability. However, AMPK activation did not directly altered Na_v_1.4 channel behaviour *in vitro*. A thorough investigation of the electrophysiological properties of the muscle fibres would be required to further characterize the possible effects of AMPK activation on muscle excitability.

In conclusion, we have identified a novel *SCN4A* mutation in a human patient and created a mouse model carrying the equivalent point mutation. Both mutations implicate a novel Na_v_1.4 domain in the pathophysiology of myotonia and periodic paralysis. In depth characterization validated the draggen mice as an excellent new model to study the underlying muscle pathophysiology of the human conditions, and uncovered novel systemic metabolic abnormalities. These metabolic alterations are accompanied by changes in muscle AMPK activation levels, suggesting a potential role of the energy sensor AMPK in models of *SCN4A* channelopathies.

## Abbreviation

AMPK: AMP-activated protein kinase
